# Effect of Boiling on *Meretrix lyrata* Protein Structure Variation and Its Mechanism Based on Data-Independent Acquisition (DIA) Quantitative Proteomics

**DOI:** 10.3390/foods14244278

**Published:** 2025-12-12

**Authors:** Wen-Hui Shang, Zhi-Chao Chang, Yi-Wen Wang, Quzhen Luosang, Yu-Yu Hang, Zhi-Sheng Pei, Xuan-Ri Shen

**Affiliations:** 1 College of Food Science and Engineering, Hainan Tropical Ocean University, Sanya 572022, China; wenhui_shang@126.com (W.-H.S.);; 2 Yazhou Bay Innovation Institute, Hainan Tropical Ocean University, Sanya 572025, China; 3 Hainan Provincial Academician Team Innovation Center, Hainan Tropical Ocean University, Sanya 572022, China; 4 Marine Food Engineering Technology Research Center of Hainan Province, Hainan Tropical Ocean University, Sanya 572022, China

**Keywords:** *Meretrix lyrata*, boiling process, protein degradation, structural dynamics, DIA proteomics

## Abstract

This study employed a multi-technique approach to investigate the structural and conformational changes in proteins in *Meretrix lyrata* (*M. lyrata*) adductor, foot, and siphon tissues during boiling. Data-independent acquisition (DIA) quantitative proteomics was utilized to identify differentially expressed proteins (DEPs) in six temporal comparison groups (20–0 s, 40–20 s, 60–40 s, 80–60 s, 100–80 s, and 120–100 s). The results showed that key myofibrillar proteins, including myosin heavy chain, paramyosin, and actin, exhibited tissue-specific expression patterns, while low-molecular-weight degradation fragments (<17 kDa) appeared with prolonged heating. Turbidity measurements peaked in adductor and siphon tissues at 60 s and in foot tissue at 80 s. Heating resulted in a narrowed particle size distribution (100–1000 nm), and a decreased zeta potential, indicating a reduction in protein surface charge. Fourier transform infrared spectroscopy revealed hydrogen bond disruption and secondary structure transitions, marked by a reduction in α-helix content with a corresponding increase in β-sheet and random coil structures. In total, 6527 proteins were identified, and Gene Ontology (GO) enrichment analysis highlighted the DEPs’ involvement in biological regulation and metabolic processes. Collectively, these results provide comprehensive characterization of protein denaturation, degradation, and structural reorganization in *M. lyrata* tissues during the boiling process.

## 1. Introduction

Hard clam proteins exhibit high digestibility and biological value due to their essential amino acid (EAA) composition, which closely aligns with the EAA requirement pattern recommended by the World Health Organization (WHO) [1]. *Meretrix lyrata* (*M. lyrata*) is an edible clam distributed throughout the Indo-West Pacific region, including the intertidal flats of China, Indonesia, and the Philippines. This species is of considerable commercial importance due to its favorable nutritional profile, with muscle protein content of 34.15% [2]. As such, *M. lyrata* represents a valuable and significant source of dietary protein.

Clam muscle tissues exhibit structural and functional heterogeneity, comprising striated, obliquely striated, and smooth muscle types [3]. In the Asian hard clam (*Meretrix lusoria*), myofibrillar proteins constitute the predominant protein fraction in the foot and mantle tissues [4]. The amino acid profile of *Paphia undulata*, which includes 21 amino acids, was reported to be comparable to those of grass carp (*Ctenopharyngodon idella*) and shrimp (*Metapenaeus ensis*), suggesting compositional parallels among aquatic species [5].

Boiling, a common thermal processing method, is known to induce extensive protein denaturation and degradation. For instance, heating surf clam (*Spisula sachalinensis*) at 80 °C would alter its microstructure and modify the proteins’ secondary and tertiary structures [6,7]. Additionally, boiling could enhance the nutritional value, digestibility, and sensory characteristics of aquatic products. High water content promotes heat penetration and facilitates protein denaturation during thermal processing [8]. As heating progresses, the hydrogen bonds and electrostatic interactions weaken, exposing the hydrophobic groups in proteins [7], and thereby altering their functional properties. However, there is a lack of research investigating on the protein structural dynamics in *M. lyrata* during cooking.

Data-independent acquisition (DIA) quantitative proteomics is a proteomic technology that focuses on comprehensive protein detection and quantification. It is a powerful method that has been used extensively for proteomic studies in aquatic animals, including silver carp (*Hypophthalmichthys molitrix*), Antarctic krill (*Euphausia superba*), and razor clam (*Sinonovacula constricta*) [9,10,11]. Hence, DIA quantitative proteomics provides an effective platform for characterizing protein changes in aquatic animals during processing.

In this study, we aim to investigate the protein dynamics in *M. lyrata* during boiling. Specimens were boiled for 20, 40, 60, 80, 100, and 120 s to obtain samples at different time points. Protein structural characterization was performed using sodium dodecyl sulfate-polyacrylamide gel electrophoresis (SDS-PAGE), turbidity, surface hydrophobicity, particle size, zeta potential, and Fourier transform infrared spectroscopy (FTIR). Subsequently, DIA quantitative proteomics was employed to identify differentially expressed proteins (DEPs) and to elucidate changes in protein composition throughout the boiling process. The integration of structural and proteomic analyses provides a comprehensive understanding of heat-induced protein dynamics in *M. lyrata*. This study not only addresses the current knowledge gap regarding the effects of boiling on *M. lyrata* proteins, but also provides essential insights into optimizing thermal processing strategies in shellfish, with the aim of preserving nutritional and sensory quality.

## 2. Materials and Methods

### 2.1. Materials and Chemicals

Live clams (*M. lyrata*) with a mean weight of 13.90 ± 1.79 g were purchased from the Nanxin Farmers’ Market in Sanya, China. The reagents 8-Anilino-1-naphthalenesulfonic acid (ANS) and potassium bromide (spectroscopic-grade) were obtained from Shanghai Macklin Biochemical Co., Ltd. (Shanghai, China). HPLC-grade formic acid and acetonitrile were obatined from Sigma-Aldrich (St. Louis, MO, USA). The Bradford protein assay kit was purchased from Takara Biomedical Technology (Beijing) Co., Ltd. (Beijing, China). The BeyoGel™ Elite pre-cast PAGE gels (Tris-Gly, 12%) and 5× SDS-PAGE sample loading buffer were purchiased from Beyotime Institute of Biotechnology (Shanghai, China). The ColorMixed Protein Marker (11–245 kDa) was purchased from Beijing Solarbio Science & Technology Co., Ltd. (Beijing, China). All other chemicals were of analytical grade and purchased from Sinopharm Chemical Reagent Co., Ltd. (Shanghai, China).

### 2.2. Sample Preparation

Live *M. lyrata* clams were boiled for 20 s, 40 s, 60 s, 80 s, 100 s, and 120 s in boiling water and then immediately transferred to an ice-water bath. Adductor muscle, foot, and siphon tissues were collected from both live and boiled clam samples for comparative analyses. All tissue samples were subsequently lyophilized. For each treatment group, tissues from six clams were pooled, mixed, and homogenized to obtain a composite experimental sample.

### 2.3. Determination of SDS-PAGE Pattern

Protein profiling was carried out based on a modified method described by Shang et al. [12]. In brief, 0.5 g of lyophilized tissue powder was mixed with 9 mL of solubilization buffer containing 0.1 M Tris-HCl (pH 8.0), 8 M urea, 4% SDS, 2% mercaptoethanol, and 20% glycerol. The mixture was boiled for 10 min and sonicated for 2 min to disrupt tissue structure. After centrifugation at 16,000× *g* for 10 min, an aliquot of 8 μL of the supernatant was subjected to SDS-PAGE using a 4% (*w*/*v*) stacking gel and a 12% (*w*/*v*) separating gel on a Bio-Rad electrophoresis system (Bio-Rad, Hercules, CA, USA). After electrophoresis, the gel was stained overnight at room temperature with 0.05% Coomassie Brilliant Blue R-250 in 50% ethanol and 7.5% acetic acid. The gel was then destained with a solution of 50% ethanol and 9% acetic acid, rinsed thoroughly with distilled water, and imaged immediately.

### 2.4. Determination of Turbidity

Turbidity was measured according to a modified method reported by Cai et al. [13]. Protein samples were suspended in 0.2 mol/L phosphate buffer (pH 7.0) and centrifuged at 4000× *g* for 15 min at 4 °C. The protein concentration in the supernatant was quantified using a Bradford Protein Assay Kit, and all samples were subsequently adjusted to an equivalent protein concentration. For turbidity measurements, a 3 mL aliquot of each solution was transferred to a quartz cuvette with a 1 cm path length. Absorbance was recorded at 660 nm using a UV-Vis spectrophotometer (Hitachi, U-2700, Tokyo, Japan) with phosphate-buffered saline (PBS) serving as the blank control.

### 2.5. Determination of Surface Hydrophobicity (H_0_)

Surface hydrophobicity was determined as described by Shang et al. [12]. Protein solutions (0.0002–0.0005 mg/mL) were prepared by dissolving samples in 10 mM phosphate buffer (pH 7.0). To each solution, 20 μL of 8 mmol/L ANS in 10 mmol/L phosphate buffer (pH 7.0) was added. Fluorescence intensity was then measured using a spectrofluorometer (Cary Eclipse, Agilent, Santa Clara, CA, USA) at excitation and emission wavelengths of 390 nm and 470 nm, respectively. The H_0_ was calculated from the slope of the fluorescence intensity versus protein concentration plot (*R*^2^ > 0.95).

### 2.6. Determination of Particle Size and Zeta Potential

Particle size and zeta potential were measured according to the method by Liu et al. [14] with minor modifications. Briefly, 0.2 g of lyophilized protein powder was dissolved in 20 mL of 10 mM phosphate buffer (pH 7.0). The mixture was stirred for 1 h and centrifuged at 4000× *g* for 15 min at 4 °C. The resulting supernatant was diluted to a protein concentration of 0.5 mg/mL, and a 1 mL aliquot was transferred to a measurement cuvette. Particle size and zeta potential were then determined using a particle size analyzer (Zetasizer Pro, Malvern, Worcestershire, UK) at 25 °C following a 120 s equilibration time.

### 2.7. Determination of Secondary Structure

The FTIR spectra of *M. lyrata* adductor, foot, and siphon proteins were recorded using a modified method described by Agbaje et al. [15]. Briefly, 2 mg of lyophilized tissue powder was ground thoroughly with 200 mg of spectroscopic-grade KBr. The resulting mixture was then compressed into a pellet and analyzed using an FTIR spectrometer (ALPHA II, Bruker, Billerica, MA, USA). Spectra processing included Fourier self-deconvolution and second-derivative analysis within the Amide I region (1700–1600 cm^−1^). PeakFit software (version 4.12, Systat Software, Inc., Richmond, VA, USA) was used to quantify the relative proportions of α-helix, β-sheet, β-turn, and random coil structures based on peak area integration [16].

### 2.8. Proteomics of Proteins in M. lyrata Clam During the Boiling Process

#### 2.8.1. Protein Digestion

Protein digestion was performed in accordance with the Filter-Aided Sample Preparation (FASP) method described by Wiśniewski et al. [17]. First, samples were mixed with an equal volume of 100 mM dithiothreitol (DTT) and boiled for 5 min. After cooling to room temperature, the mixtures were transferred to 10 kDa ultrafiltration centrifuge tubes (Millipore Amicon Ultra-0.5, Billerica, MA, USA) and were washed twice with 200 μL of uric acid (UA) buffer (8 M urea, 150 mM Tris-HCl, pH 8.0) by centrifugation at 12,000× *g* for 15 min, with the supernatant discarded after each wash. The retentate was then incubated with 100 μL of 50 mM iodoacetamide (IAA) in UA buffer for 20 min in the dark with gentle shaking (600 rpm), before being centrifuged at 12,000× *g* for 30 min. Subsequently, the retentate was washed twice with 100 μL of UA buffer and centrifuged at 12,000× *g* for 10 min each time. The retentate was then resuspended in 100 μL of 50 mM NH_4_HCO_3_ buffer and centrifuged at 14,000× *g* for 10 min, and this wash step was repeated twice.

Protein hydrolysis was performed by resuspending the retentate in 40 μL of trypsin solution (6 μg trypsin in 40 μL of 50 mM NH_4_HCO_3_ buffer) with shaking at 600 rpm for 1 min, followed by incubation at 37 °C for 16–18 h. The resulting peptide supernatant was collected by centrifugation at 12,000× *g* for 10 min at 25 °C. The supernatant was acidified with trifluoroacetic acid (TFA) to a final concentration of 0.1%, desalted using a Sep-Pak C18 cartridge (Waters, Milford, MA, USA), and dried in a vacuum concentrator. The resulting peptide powder was reconstituted in 20 μL of 0.1% formic acid (FA) for Liquid Chromatography–Tandem Mass Spectrometry (LC-MS/MS) analysis.

#### 2.8.2. LC-MS/MS Analysis for DIA

Peptide samples were analyzed according to Jiao et al. [9] with minor modifications. Separation was performed on a Vanquish Neo Ultra-High Performance Liquid Chromatography (UHPLC) system coupled to an Orbitrap Astral mass spectrometer (Thermo Fisher Scientific, Bremen, Germany). Peptides were loaded onto a 50 cm µPAC™ Neo column (Thermo Fisher Scientific, Bremen, Germany) at a flow rate of 2.2 μL/min. The reversed-phase High-Performance Liquid Chromatography (RP-HPLC) mobile phases consisted of: Phase A, 0.1% FA; and Phase B, 0.1% FA in 80% acetonitrile. The linear gradient program was as follows: 0–0.1 min, 4–6% B; 0.1–1.1 min, 6–12% B; 1.1–4.3 min, 12–25% B; 4.3–6.1 min, 25–45% B; 6.1–6.5 min, 45–99% B; 6.5–8.0 min, 99% B.

Data acquisition was performed in DIA mode using Xcalibur 7.5.1 (Thermo Fisher Scientific, Bremen, Germany). The acquisition parameters were: total run time, 8 min; spray voltage, 2.2 kV; polarity, positive; MS1 scan range, 380–980 *m*/*z*. MS1 resolution was set to 240,000 at *m*/*z* 200 with an automatic gain control (AGC) target of 500% and a maximum injection time of 3 ms. The MS2 resolution was set to 80,000 with an AGC target of 500%, maximum injection time 0.6 s, RF lens at 40%, higher-energy collisional dissociation (HCD) activation, a 2 Th isolation window, and normalized collision energy of 25%. Both MS1 and MS2 spectra were acquired in profile mode.

#### 2.8.3. DIA Sequence Database Searching

The DIA data processing and protein quantification were performed using DIA-NN 1.8.1 according to approaches as described by Demichev et al. [18] and Barkovits et al. [19]. The MS data were searched against the verified protein sequences of *M. lyrata* in the UniProtKB/Swiss-Prot database (https://www.expasy.org/resources/uniprotkb-swiss-prot; accessed on 1 March 2025). The database search parameters were as follows: (1) a maximum of one missed cleavage site; (2) precursor mass tolerance of 10 ppm; (3) fragment mass tolerance of 10 ppm. Trypsin was specified as the digestion enzyme. carbamidomethylation (C) was set as a fixed modification, while N-terminal acetylation and oxidation (M) were set as variable modifications with a maximum of 1 variable modification allowed per peptide. Peptide length was set to 7–30 amino acids, and charge states +1 to +4 were considered. Fragment ion *m*/*z* range was set to 150–2000 in centroid mode. Search results were filtered to a 1% false discovery rate (FDR)at both peptide-spectrum match (PSM) and protein levels. All procedures followed established proteomics protocols without specific technical standard citations.

### 2.9. Bioinformatic and Statistical Analysis

Six comparison groups (20–0 s, 40–20 s, 60–40 s, 80–60 s, 100–80 s and 120–100 s) were analyzed for protein quantification. The DEPs were defined as those showing a fold change (FC) > 1.5 or <0.67 combined with Student’s *t*-test *p* < 0.05 [9]. Statistical analyses were performed using R version 4.3.0 (R Foundation for Statistical Computing, Vienna, Austria) and Microsoft Excel 2019 (Microsoft Corporation, Redmond, WA, USA). Hierarchical clustering and volcano plots were generated using R software. Protein sequences were annotated against the UniProtKB/Swiss-Prot database (https://www.expasy.org/resources/uniprotkb-swiss-prot; accessed on 1 March 2025) and Gene Ontology (GO) (http://geneontology.org; accessed on 1 March 2025) resource [20]. The GO enrichment analysis was performed using Fisher’s exact test with the entire identified proteome as background, followed by Benjamini–Hochberg false discovery rate (FDR) correction. Enriched GO terms were considered significant at an FDR-adjusted *p* < 0.01.

All experiments were performed using three biological replicates, and the results are expressed as mean ± standard deviation (SD). Statistical significance was determined by one-way ANOVA followed by Duncan’s multiple range test (*p <* 0.05) using IBM SPSS Statistics version 27.0.0 (IBM Corporation, Armonk, NY, USA).

## 3. Results and Discussion

### 3.1. Changes in SDS-PAGE Pattern

**Figure 1:**
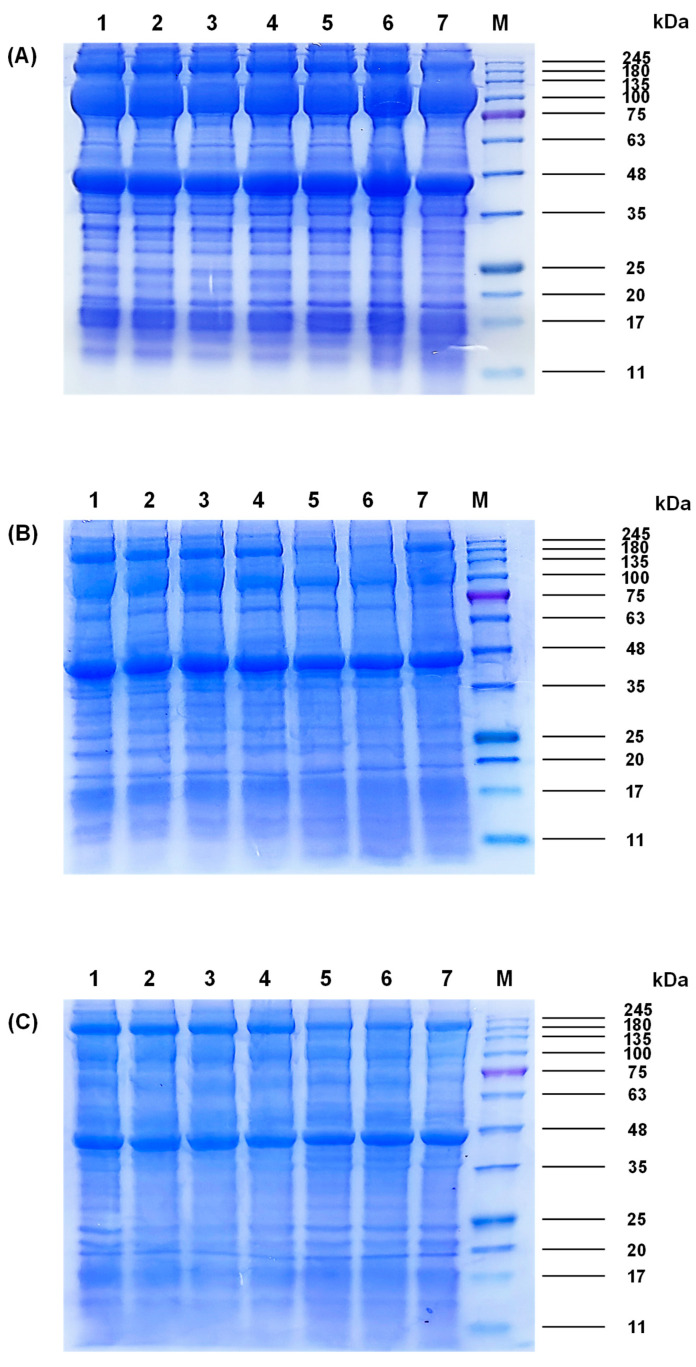
SDS-PAGE patterns of different fractions from the (**A**) adductor, (**B**) foot, and (**C**) siphon of *M. lyrata*. Samples in lanes 1, 2, 3, 4, 5, 6, and 7 represented boiling times of 0 s, 20 s, 40 s, 60 s, 80 s, 100 s and 120 s, respectively. M denoted the molecular weight standard.

Protein profiles of *M. lyrata* adductor, foot, and siphon tissues are shown in Figure 1. Myosin heavy chain (MHC, ~200 kDa) and actin (~42 kDa) were ubiquitously expressed across all three tissues, whereas PA (~100 kDa) exhibited tissue-specific distribution, being abundant in adductor and foot but barely detectable in the siphon tissues (Figure 1).

SDS-PAGE analysis revealed that MHC from *M. lyrata* adductor, foot, and siphon migrated at ~200 kDa (Figure 1), consistent with the reported MHC molecular weight of ~210 kDa in baby clam (*Paphia undulata*) [4]. Paramyosin (PA) was detected at ~100 kDa in all tissues, which is aligned with observations in the Asian hard clam (*Meretrix lusoria*) where PA similarly migrates at ~100 kDa [4]. Actin (~45 kDa) was ubiquitously expressed across all tissues (Figure 1), consistent with results reported in *Mactra chinensis* tissue [21].

Quantitative densitometry further revealed tissue-specific expression levels: band intensities of MHC and actin were highest in the adductor, followed by the foot, and were least abundant in the siphon (Figure 1). Low-molecular-weight fragments (<17 kDa) emerged after boiling the adductor for 100–120 s (Figure 1A) and after 80–120 s for the foot and siphon (Figure 1B,C), indicating a progressive protein degradation.

Shellfish proteins generally consist of water-soluble, salt-soluble, and insoluble fractions. Salt-soluble proteins such as MHC, myosin light chain (MLC), actin, and tropomyosin typically contribute to muscle contraction. Previous studies on limpet (*Patella vulgata*) and *M. lusoria* have revealed variations in water-soluble and salt-soluble protein composition [22,23], which may be attributed to muscle functions and structural organization. A well-balanced distribution of protein types is essential for optimal contraction and metabolism in molluscan muscles [24]. Furthermore, the major water-soluble proteins in the scallop adductor muscle are reported to be mainly distributed in the range between 10 and 250 kDa [25]. During boiling, thermal-induced protein denaturation in *M. lyrata* tissues led to decreased protein solubility associated with structural alterations. Similar reductions in tissue solubility caused by leaching of water-soluble proteins have been documented in Asiatic hard clam (*Meretrix meretrix*) during heat treatment [26].

According to Tang et al. [23], the insoluble protein fractions in *M. lusoria* exhibit molecular weights ranging from 44.3 to 200 kDu, which affects their solubility in the loading buffer. Therefore, the bands observed in lanes 5 and 6 of Figure 1B, migrating at approximately 200 kDa, are most likely attributable to these insoluble proteins. This interpretation is further supported by their reduced band intensities, which suggested low protein solubilities. These findings indicate that the thermal treatment has altered the protein composition. Taken together, boiling-induced protein degradation and denaturation contributed to structural changes in *M. lyrata* proteins.

### 3.2. Changes in Protein Turbidity

**Figure 2:**
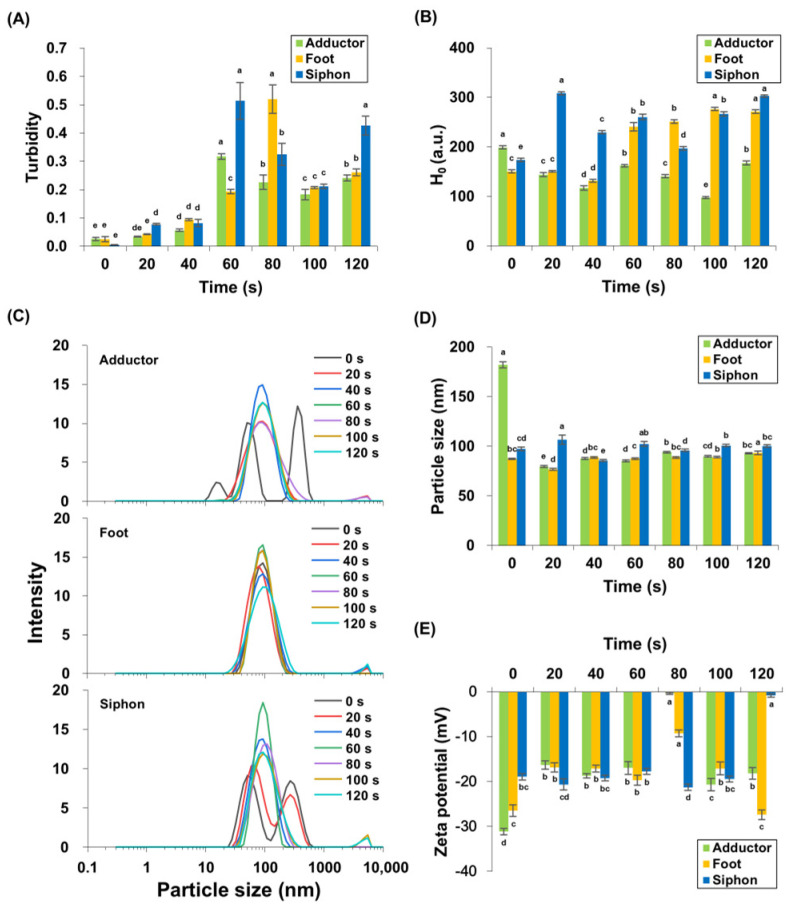
Turbidity (**A**), surface hydrophobicity (**B**), particle size distribution (**C**,**D**), and zeta potential (**E**) were measured in *M. lyrata* adductor, foot, and siphon tissues during the boiling process. Different letters indicate significant differences (*p* < 0.05).

Protein turbidity of *M. lyrata* adductor, foot, and siphon tissues varied during boiling (Figure 2A). Throughout the boiling process, turbidity exhibited a significant (*p* < 0.05) triphasic pattern, characterized by an initial increase, followed by a decrease, and a subsequent rise. Baseline turbidity was lowest in unheated samples, reflecting minimal protein aggregation. After 60 s of boiling, the adductor and siphon tissues achieved peak protein turbidity values of 0.32 ± 0.01 and 0.51 ± 0.07, respectively, with no significant difference in siphon turbidity between 60 s and 120 s (*p* > 0.05). In contrast, the foot tissues exhibited a maximum protein turbidity of 0.52 ± 0.05 after 80 s of boiling. These findings are consistent with observations by Dong et al. [27], who found that the turbidity of scallop (*Patinopecten yessoensis*) actomyosin solution increased upon heating from 20 to 90 °C. Similarly, Reed and Park [28] reported that the turbidity of tilapia myosin increased at 39 °C, plateaued, and stabilized between 70 and 90 °C. In *M. lyrata*, the initial turbidity increase likely reflects heat-induced protein unfolding and aggregation, while the subsequent decrease after 60–80 s (Figure 2A) may result from flocculation and coalescence of protein aggregates [28]. Results from SDS-PAGE analysis confirmed thermal degradation across all tissues (Figure 1), with protein bands ranging from 25–210 kDa contributing to the sample turbidity (Figure 1). The formation of high-molecular-weight aggregates during boiling further increased the turbidity. Akihiro et al. [29] identified a 33 kDa protein in the boiled soup of bloody clam (*Anadara broughtonii*), Asiatic hard clam (*Meretrix lusoria*), Mediterranean mussel (*Mytilus galloprovincialis*), and other similar species, supporting the notion that protein aggregation during thermal processing is a common phenomenon in bivalve tissues.

### 3.3. Changes in Surface Hydrophobicity (H_0_)

Surface hydrophobicity was measured to evaluate protein conformation, as indicated by exposed hydrophobic group content. Figure 2B shows that *M. lyrata* adductor, foot, and siphon tissues exhibited dynamic changes in surface hydrophobicity during boiling. The H_0_ in *M. lyrata* foot tissue significantly increased from 131.70 ± 3.53 to 271.64 ± 2.63 between 40 s and 120 s (*p* < 0.05). Similarly, H_0_ in siphon samples rose significantly from 80 to 120 s of boiling (*p* < 0.05), while adductor H_0_ showed a significant increase at 100 s and 120 s (*p* < 0.05). Overall, H_0_ increased significantly in all tissues by the end of boiling (*p* < 0.05).

These observations are consistent with trends reported in ready-to-eat shrimp boiled at 121 °C for 0, 2, 4, and 6 min [30]. The distinct protein compositions of *M. lyrata* adductor, foot, and siphon tissues (Figure 1) likely contributed to the tissue-specific changes in surface hydrophobicity. During boiling, protein unfolding exposed buried hydrophobic groups, increasing H_0_, whereas self-assembly and aggregation partially buried these groups, reducing H_0_ [31,32]. As boiling time progressed, conformational alterations and other structural modifications redistributed hydrophobic groups on the protein surface, resulting in the observed dynamic changes in H_0_.

### 3.4. Changes in Particle Size and Zeta Potential

Particle size distribution reflected the degree of protein aggregation and cross-linking. Figure 2C,D show that the particle size distributions and volume-weighted mean diameters for boiled *M. lyrata* adductor, foot, and siphon tissues were mainly distributed in the 100–1000 nm range, while untreated samples of *M. lyrata* adductor and siphon tissues exhibited a relatively broad particle size distribution. The fresh adductor exhibited a trimodal particle size distribution, peaking at 182.23 ± 3.05 nm, whereas both fresh and 20 s boiled siphon samples showed bimodal distributions. Boiling narrowed the distribution to a single peak at ~150 nm (Figure 2C). These results are in agreement with the findings by Chen et al. [33], which indicated that untreated, aggregated tropomyosin exhibited the largest particle size among samples. Since high-temperature cooking can simultaneously induce both protein aggregation and particle size reduction [34,35], the observed decreases in peak intensity and particle size of *M. lyrata* adductor and siphon tissues are thus likely to reflect molecular structural changes during boiling, resulting in altered protein dimensions. In contrast, foot tissue displayed a single peak (Figure 2C), indicating relatively stable protein particle size throughout boiling. Low-intensity peaks in the 1000–10,000 nm range were also detected, which may be due to aggregation of small particles and protein crosslinking, as reported for Antarctic krill proteins [32].

Zeta potential describes the distribution of net charge on the protein surface, with higher absolute values indicating stronger electrostatic repulsions between particles [36]. Figure 2D shows the zeta potential variations in *M. lyrata* adductor, foot, and siphon protein particles during boiling. Our results indicate that the zeta potential of adductor and foot protein particles initially increased and then decreased, reaching minimum absolute zeta potentials of −0.39 ± 0.19 mV and −9.26 ± 0.79 mV, respectively, at 80 s. In siphon tissues, the zeta-potential of the proteins decreased, from approximately −20 mV to −0.8 ± 0.36 mV (Figure 2D) following a 120 s boiling treatment. These trends are similarly reported for bighead carp myosin, whose zeta potential decreases with prolonged boiling time [37]. The negative zeta potential is primarily attributed to the exposure of anionic amino acid residues on its surface [38], and may also reflect the myosin content in *M. lyrata* tissues. As reported by Sun et al. [39], protein dispersions generally achieve greater stability when the absolute zeta potential value exceeds 20 mV. However, as thermal treatment induces the aggregation of myosin particles, it may obscure the negatively charged amino acid side chains and alter the protein surface charge, ultimately leading to a reduction in the absolute zeta potential value [40,41].

### 3.5. Changes in Secondary Structure

**Figure 3:**
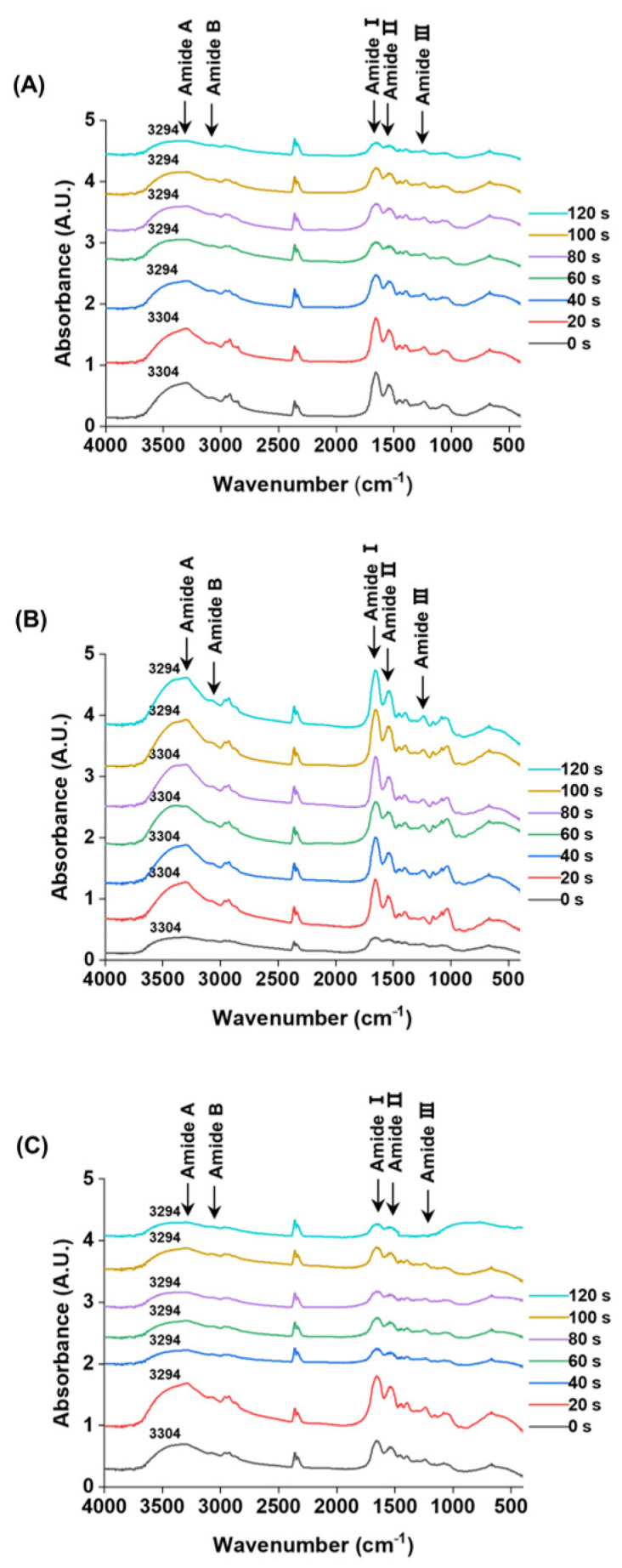
FTIR spectra of the *M. lyrata* adductor (**A**), foot (**B**), and siphon (**C**) tissues during the boiling process.

Fourier-transform infrared (FTIR) spectroscopy is a technique that probes molecular vibrations through infrared absorption, allowing the identification of functional groups based on their characteristic vibrational modes [42]. The FTIR spectra of *M. lyrata* adductor, foot, and siphon tissues are presented in Figure 3, providing insights into their protein secondary structures. Comparison of SDS-PAGE profiles revealed no major changes in protein band patterns between treated and untreated samples (Figure 1).

Although all samples exhibited absorption in the amide region (400–4000 cm^−1^), the three tissue types nevertheless displayed distinct spectral intensities and band shifts during boiling. Specifically, the Amide A band shifted from approximately 3294 to 3304 cm^−1^, with the timing and extent of this shift varying among tissue types (Figure 3). Such shifts in the Amide A band are indicative of increased hydrogen bonding involving N-H groups [43]. The characteristic Amide B bands appeared in the range of 3076.0–3090.5 cm^−1^; for instance, the adductor tissue exhibited a band at 3084.3 cm^−1^. These vibrations are typically associated with the asymmetric stretching of =C-H and -NH_3_^+^ groups, as reported for gelatin from the swim bladder of yellowfin tuna [44].

The characteristic Amide I, II, and III bands were detected in the ranges of 1650.2–1658.4 cm^−1^, 1530.9–1547.3 cm^−1^, and 1234.6–1238.7 cm^−1^, respectively (Figure 3). These bands arise from specific atomic displacements: Amide I (~1650 cm^−1^), Amide II (~1550 cm^−1^), and Amide III (~1300 cm^−1^) involve a combination of in-plane (e.g., C=O stretching, C-N stretching, N-H bending) and out-of-plane motions (e.g., C-N torsion, C=O bending) [45,46].

During boiling, a blue shift in the Amide I band was observed for the adductor (from 1650.2 to 1656.4 cm^−1^) and foot (from 1654.3 to 1656.4 cm^−1^) tissues. In contrast, the siphon tissue displayed both a blue shift and peak splitting: a single peak at 1652.2 cm^−1^ resolved into a doublet at 1656.4 cm^−1^ and 1650.2 cm^−1^ (Figure 3). This phenomenon is suggestive of protein denaturation, wherein the native peak at ~1651 cm^−1^ (associated with α-helical structures) diminishes as a new peak at ~1658 cm^−1^ (corresponding to denatured protein) emerges, as reported in hemoglobin studies [47].

The Amide II bands of the *M. lyrata* adductor tissue were observed within the range of 1537.0–1547.3 cm^−1^. In contrast, the foot and siphon tissues each exhibited two distinct peaks at 1547.3 cm^−1^ and 1537.0 cm^−1^ after boiling for 20 s and 40 s, respectively (Figure 3). This splitting is consistent with previous findings for egg white proteins, which displayed two peaks at 1545 cm^−1^ and 1535 cm^−1^. The ~10 cm^−1^ red-shift is primarily attributed to the disruption of hydrogen bonds, which restricts protein refolding [48]. Meanwhile, the Amide III bands for all three tissues (adductor, foot, and siphon) were detected in the range of 1234.6–1238.7 cm^−1^. These vibrations arise from a combination of C–N stretching and N–H deformation in the amide linkage, with additional contributions from CH_2_ wagging vibrations in the glycine backbone and proline side chains [49].

The secondary structure composition (α-helix, β-sheet, β-turn, and random coil) of proteins in *M. lyrata* adductor, foot, and siphon tissues was quantified from the infrared spectra in the 1600–1700 cm^−1^ region using Peak Fit software. As summarized in Table 1, the protein secondary structure was progressively altered with increasing boiling time. Initially, the α-helix was the predominant conformation in all tissues. Over time, thermal denaturation of proteins occurred, as characterized by a marked decrease in α-helix content, coupled with a concurrent increase in β-sheet and random coil structures. This transformation reflects the conversion of ordered, rigid secondary structures into more disordered and flexible conformations upon heating, which can be attributed to thermal disruption of hydrogen bonds, destabilizing native structures while promoting protein unfolding, particularly at exposed hydrophilic residues and flexible loops [50]. Additionally, protein leaching from the tissues may have further contributed to these changes. Similar structural transitions have been reported for tropomyosin from the clam *Mactra veneriformis*, where combined heat and pressure treatments reduced the α-helix content [33]. Likewise, thermal dissociation of the myosin tail can induce conversion of α-helices into β-sheets [51]. Interestingly, the α-helix content in *M. lyrata* tissues exhibited an initial decrease, followed by a subsequent increase (Table 1). A comparable non-monotonic trend has been observed in beef myosin, where the α-helix content increased slightly as the temperature rose from 55 °C to 75 °C before decreasing at higher temperatures [51]. The initial increase in α-helix content may be due to the rearrangement of intramolecular hydrogen bonds, leading to a temporary stabilization of helical structures [52].

Although the adductor, foot, and siphon tissues exhibited distinct absolute secondary structure contents, they exhibited similar trends in their structural changes during boiling (Table 1). These differences are likely to stem from variations in native protein composition and conformation across tissue types. In contrast, the parallel trends in their structural shifts suggest a common underlying protein constitution and denaturation mechanism in *M. lyrata* tissues.

### 3.6. Proteome Analysis of Proteins in M. lyrata

**Figure 4:**
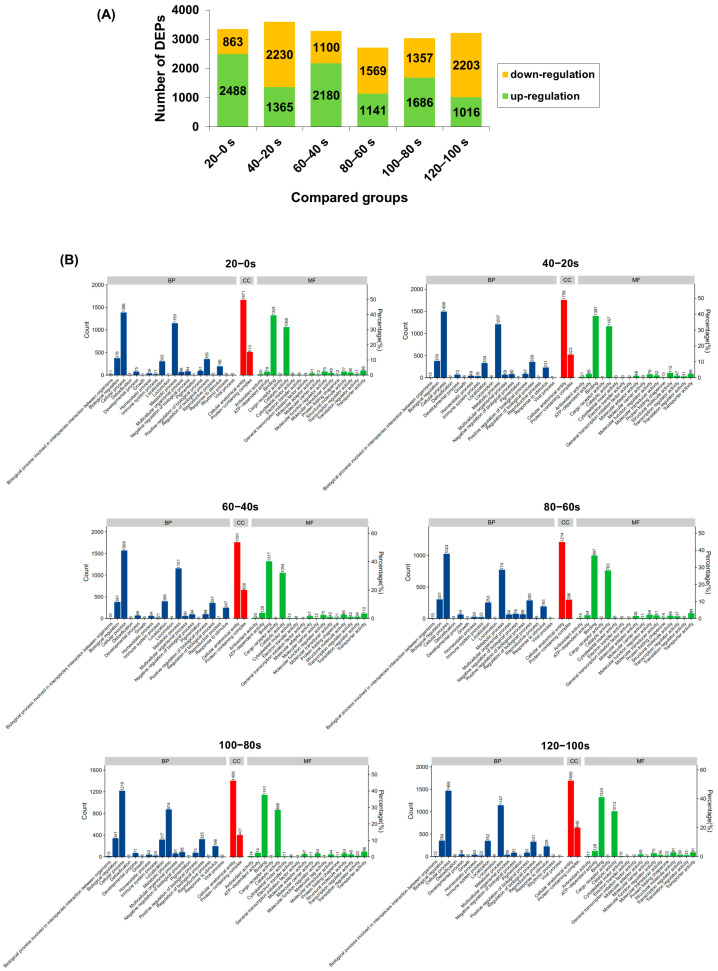
*M. lyrata* differential proteomic profile of proteins dealt with boiling process. (**A**) Count of up- and down-regulated differentially expressed proteins (DEPs) in each pairwise comparison (20–0 s, 40–20 s, 60–40 s, 80–60 s, 100–80 s and 120–100 s). (**B**) Gene Ontology (GO) classification of differentially expressed proteins (DEPs) in the following pairwise comparisons: 20–0 s, 40–20 s, 60–40 s, 80–60 s, 100–80 s and 120–100 s. BP denoted biological processes; CC, cellular components; and MF, molecular functions. DEPs were considered significantly regulated if they exhibited a fold change (FC) > 1.5 or < 0.67.

Data-independent acquisition (DIA) quantitative proteomics identified 6527 proteins from *M. lyrata* tissues. The numbers of differentially expressed proteins (DEPs) in the six pairwise comparisons (20–0 s, 40–20 s, 60–40 s, 80–60 s, 100–80 s and 120–100 s) were 3351, 3595, 3280, 2710, 3043, and 3219, respectively (Figure 4A). Notably, compared with the 20–0 s group, the number of up-regulated DEPs decreased in the subsequent comparisons (60–40 s to 120–100 s), while the number of down-regulated DEPs increased. Given that changes in protein expression during boiling are primarily attributed to thermal denaturation [53], the DEPs identified here provide a comprehensive view of proteins affected by heat treatment in *M. lyrata*.

Gene Ontology (GO) enrichment analysis was conducted on the DEPs from all six pairwise comparisons (20–0 s, 40–20 s, 60–40 s, 80–60 s, 100–80 s and 120–100 s), categorizing them into biological processes (BP), cellular components (CC), and molecular functions (MF) (Figure 4B). The majority of DEPs across all comparisons were enriched in the BP category. Within BP, the most prominent terms were “biological regulation” and “metabolic process”, which are intrinsically linked as the former can influence the latter at multiple levels [54]. In the MF category, the dominant terms were “binding” and “catalytic activity”, reflecting the interdependence of enzyme-substrate interactions for catalysis [55]. Conversely, the CC category contained the fewest DEPs; among these, “cellular anatomical entity” was the most represented, encompassing a higher number of DEPs than other CC terms, such as “protein-containing complex” (Figure 4B). Collectively, these GO results indicate that boiling significantly affects proteins involved in metabolic and regulatory functions in *M. lyrata* tissues.

**Figure 5:**
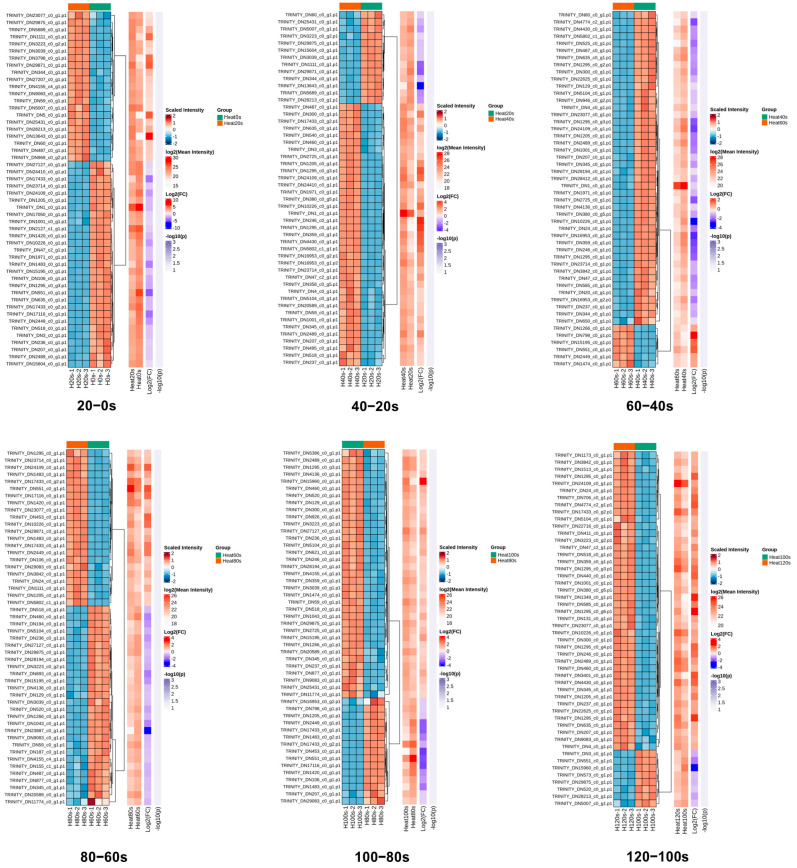
Hierarchical cluster analysis of the quantified proteins in 20–0 s, 40–20 s, 60–40 s, 80–60 s, 100–80 s and 120–100 s compared groups. Relative expression levels are represented by a color gradient, with red denoting up-regulation and blue denoting down-regulation.

Hierarchical cluster analysis was employed to examine the patterns of DEPs across the six pairwise comparisons (20–0 s, 40–20 s, 60–40 s, 80–60 s, 100–80 s and 120–100 s). A heatmap was constructed to visualize the expression profiles of the top 50 DEPs with the largest fold changes, using Euclidean distance as the clustering metric (Figure 5). The high consistency among biological replicates within each group underscores the reliability of the dataset.

The clustering revealed dynamic changes in protein expression during boiling. The number of up-regulated DEPs initially increased, then decreased, and subsequently increased again across the sequential comparisons. Conversely, the down-regulated DEPs displayed an inverse pattern (Figure 5). These oscillating shifts in protein expression reflect the progressive denaturation and complex transformation of protein characteristics induced by the boiling process. Such dynamic responses of DEPs during thermal processing align with previous findings in boiled abalone [53], highlighting the intricate nature of heat-induced modifications in shellfish proteomes.

## 4. Conclusions

This study systematically elucidated the structural and proteomic changes in *Meretrix lyrata* tissues during boiling. Thermal treatment induced tissue-specific protein denaturation and degradation, as evidenced by SDS-PAGE and the emergence of low-molecular-weight fragments. Turbidity, surface hydrophobicity, particle size, and zeta potential analyses revealed distinct aggregation and unfolding kinetics, indicating progressive structural destabilization and diminished electrostatic repulsion. FTIR spectroscopy demonstrated significant secondary structure transitions, including decreases in α-helix content and increases in β-sheet and random coil structures, reflecting disrupted hydrogen bonding and impaired refolding capacity. DIA-based quantitative proteomics identified 6527 proteins, with differentially expressed proteins predominantly involved in metabolic and regulatory processes, highlighting the broad functional impact of boiling. Collectively, these findings provide a comprehensive understanding of heat-induced protein dynamics in *M. lyrata* and offer a scientific foundation for optimizing thermal processing to preserve the nutritional and textural qualities of shellfish products. Limitations regarding absolute quantification and distinction between intact proteins and degradation products suggest that future studies could employ techniques such as TMT or iTRAQ for more precise protein quantification.

## Figures and Tables

**Table 1:** Effects of heating time on secondary structure of *M. lyrata* adductor, foot and siphon protein.

Sample	Time (s)	*α*-Helix (%)	*β*-Sheet (%)	*β*-Turn (%)	Random Coil (%)
Adductor	0	49.49 ± 0.19 ^a^	18.35 ± 0.12 ^f^	31.89 ± 0.22 ^d^	0.27 ± 0.16 ^g^
	20	48.14 ± 0.24 ^b^	19.26 ± 0.17 ^e^	30.81 ± 0.11 ^e^	1.79 ± 0.19 ^f^
	40	30.93 ± 0.21 ^c^	25.02 ± 0.07 ^d^	41.51 ± 0.14 ^c^	2.54 ± 0.02 ^e^
	60	21.32 ± 0.14 ^d^	26.33 ± 0.15 ^c^	46.73 ± 0.07 ^a^	5.62 ± 0.07 ^d^
	80	8.97 ± 0.32 ^f^	34.87 ± 0.25 ^b^	43.43 ± 0.09 ^b^	12.73 ± 0.13 ^c^
	100	12.82 ± 0.21 ^e^	49.73 ± 0.47 ^a^	24.35 ± 0.04 ^f^	13.10 ± 0.06 ^b^
	120	3.46 ± 0.03 ^g^	48.66 ± 0.81 ^a^	31.88 ± 0.26 ^d^	16.00 ± 0.15 ^a^
Foot	0	47.02 ± 0.07 ^a^	14.69 ± 0.25 ^g^	37.23 ± 0.15 ^b^	1.06 ± 0.02 ^g^
	20	47.48 ± 0.17 ^a^	18.73 ± 0.21 ^f^	30.26 ± 0.09 ^d^	3.53 ± 0.02 ^f^
	40	41.78 ± 0.19 ^b^	36.04 ± 0.05 ^e^	17.95 ± 0.09 ^g^	4.23 ± 0.12 ^e^
	60	11.42 ± 0.14 ^d^	41.83 ± 0.22 ^d^	39.86 ± 0.14 ^a^	6.89 ± 0.16 ^d^
	80	18.25 ± 0.11 ^c^	44.18 ± 0.17 ^c^	28.33 ± 0.36 ^e^	9.24 ± 0.03 ^c^
	100	8.43 ± 0.28 ^e^	45.95 ± 0.28 ^b^	31.27 ± 0.21 ^c^	14.35 ± 0.24 ^b^
	120	6.23 ± 0.15 ^f^	51.58 ± 0.33 ^a^	26.05 ± 0.22 ^f^	16.14 ± 0.12 ^a^
Siphon	0	74.72 ± 0.07 ^a^	18.60 ± 0.20 ^g^	5.67 ± 0.25 ^g^	1.01 ± 0.21 ^d^
	20	55.22 ± 0.06 ^b^	24.37 ± 0.04 ^f^	19.29 ± 0.27 ^f^	1.12 ± 0.13 ^d^
	40	21.60 ± 0.20 ^c^	35.14 ± 0.12 ^e^	41.30 ± 0.29 ^a^	1.96 ± 0.21 ^c^
	60	18.73 ± 0.29 ^e^	40.89 ± 0.24 ^d^	38.37 ± 0.04 ^c^	2.01 ± 0.04 ^c^
	80	19.76 ± 0.15 ^d^	42.95 ± 0.28 ^c^	34.99 ± 0.09 ^e^	2.30 ± 0.02 ^c^
	100	10.81 ± 0.19 ^f^	45.49 ± 0.12 ^b^	40.44 ± 0.14 ^b^	3.26 ± 0.03 ^b^
	120	3.74 ± 0.14 ^g^	52.41 ± 0.09 ^a^	37.26 ± 0.03 ^d^	6.59 ± 0.19 ^a^

Values are expressed as mean ± standard deviation. Different superscript letters in the same column present statistically significant differences between boiling time (*p* < 0.05), as determined by Tukey’s multiple range test.

## Data Availability

The original contributions presented in this study are included in the article. Further inquiries can be directed to the corresponding author.
